# Virtual-reality cognitive behavior therapy versus cognitive behavior therapy for paranoid ideation: A pragmatic, single-blind, multicenter randomized clinical superiority trial

**DOI:** 10.1017/S0033291725100949

**Published:** 2025-07-04

**Authors:** Elise C.D. van der Stouwe, Chris N.W. Geraets, Maureen Berkhof, Marit Hidding, Sven van Amstel, David van den Berg, Rob van Grunsven, José de Jager, Evelien Kooijmans, Margaux Sageot, Anton B.P. Staring, Maarten Vos, Catharina E.R. Zandee, Wim Veling

**Affiliations:** 1 University Medical Center Groningen, University of Groningen, Groningen, The Netherlands; 2 GGZ Rivierduinen, Leiden, The Netherlands; 3 Mark van der Gaag Research Centre, Parnassia Psychiatric Institute, The Hague, The Netherlands; 4 Amsterdam Public Health Research Institute, Vrije Universiteit Amsterdam, Amsterdam, The Netherlands; 5 GGZ Noord-Holland-Noord, Heerhugowaard, The Netherlands; 6 Pro Persona, Arnhem, The Netherlands; 7 Center for Clinical Psychiatry, KU Leuven, Leuven, Belgium; 8 Psychologist Netherlands Mental Healthcare, Utrecht, The Netherlands; 9 GGZ Delfland, Delft, The Netherlands

**Keywords:** cognitive therapy, exposure, paranoia, psychosis, social withdrawal, virtual reality

## Abstract

**Background:**

Virtual reality (VR) may improve psychological treatments for psychotic disorders. We investigated the effects of VR-based cognitive behavior therapy for paranoid ideation (VR-CBTp) compared to standard CBTp.

**Methods:**

We conducted a pragmatic, single-blind, randomized clinical trial in seven mental health centers across the Netherlands and Belgium. A total of 98 participants with a psychotic spectrum disorder and paranoid ideation were randomized to a maximum of 16 sessions of VR-CBTp (*n* = 48) or CBTp (*n* = 50). The primary outcome was momentary paranoia, measured with the experience sampling method (ESM) at posttreatment. Secondary measures, assessed at baseline, posttreatment, and 3-month follow-up, included symptoms (paranoia, hallucination, depression, cognition, and anxiety related), social functioning, self-esteem, and schemes.

**Results:**

Both groups showed reductions in momentary paranoia between baseline and posttreatment (*n* = 56, *b* = −15.0, effect size [ES] = 0.65), but those were greater for VR-CBT (interaction *b* = 8.3, ES = 0.62). Reductions remained at follow-up (*n* = 50, *b* = −10.7, ES = 0.57) but not the interaction. Limited ESM compliance resulted in data loss; however, secondary paranoia measures did confirm improvements (ES range = 0.66–1.15, *n* = 78–81), but not the interaction. Both groups improved in symptoms, self-esteem, and social functioning. Interaction effects in favor of VR-CBTp were found for safety behavior, depression, and self-esteem at posttreatment, and self-esteem and anxiety at follow-up. For VR-CBTp, 37.5% did not complete treatment; for CBTp, this was 24.0%. Completers, on average, received 12.7 (VR-CBTp: standard deviation [SD] = 3.9) and 15.1 (CBTp: SD = 2.5) sessions.

**Conclusions:**

Both CBTp and VR-CBTp are efficacious treatments for paranoid ideation, but VR-CBTp may be somewhat more effective. Limitations concern missing primary outcome data and a lower sample size than anticipated.

## Introduction

Cognitive behavior therapy is the main evidence-based psychological treatment for paranoid ideation in patients with psychotic disorders. International treatment guidelines recommend that all patients with psychotic disorders should be offered cognitive behavior therapy for paranoid ideation (CBTp) in addition to antipsychotic medication (GGZ Standaarden, 2017; Haddock et al., 2014). The efficacy of CBTp for psychotic symptoms, however, is modest, with estimated effect sizes of 0.24–0.49 (Bighelli et al., 2018; Van der Gaag, Valmaggia, & Smit, 2014). For paranoid delusions specifically, a meta-analysis found a mean effect size of 0.27 compared to treatment as usual (Mehl, Werner, & Lincoln, 2015). New developments in CBTp interventions may enhance efficacy, such as targeting factors like worry rather than delusions directly (Freeman, Emsley, et al., 2021) or virtual reality (VR)-CBTp (Salahuddin et al., 2024). Initial randomized controlled trials (RCTs) of VR-CBTp found large effect sizes (Freeman et al., 2016; Pot-Kolder et al., 2018) but did not compare VR-CBTp with standard CBTp.

VR-CBTp may be more effective than standard CBTp because it facilitates faster and more consistent behavior interventions aimed at reducing avoidance and dropping safety behaviors, factors that have been shown to be critical for treatment effect. Specifically, VR facilitates controllable and repeated exposure on demand, lowers the threshold for patients to engage in behavior exercises, and is likely to increase the time spent on behavior interventions in sessions. According to the cognitive model of paranoia, paranoid ideation is maintained by safety behaviors such as avoidance of potentially threatening events or maladaptive coping behavior as an active effort to reduce the threat during perceived threatening events (Freeman, 2016). Therefore, dropping safety behavior is a key treatment target to reduce negative appraisal and fear by experiencing no bad consequences in perceived threatening situations. In a previous RCT of VR-CBTp by our group, a decrease in safety behavior but not cognitive biases was related to a reduction of paranoid ideation (Pot-Kolder et al., 2018). Also, safety behavior was the single moderator of the effect of VR-CBTp (Berkhof et al., 2024). Standard CBTp generally requires starting with several sessions of cognitive interventions to reduce the conviction of paranoid thoughts to enhance motivation to start behavior interventions. With the possibility to practice safely in VR environments, tailor the VR environments to individual needs, and have complete control over what happens in VR, the threshold for engaging in exposure and behavior experiments is lower in VR-CBTp.

These characteristics of VR-CBTp may not only result in a stronger effect on paranoid ideation compared to CBTp, but they may also increase treatment efficiency. The minimal treatment duration of CBTp for paranoid ideation is currently unclear. A meta-analysis on brief CBTp (defined as 6–10 sessions) reported beneficial effects and concluded that the number of sessions did not moderate treatment outcomes (Naeem et al., 2016). However, a more recent individual participant data meta-analysis demonstrated that patients who received a higher number of CBTp sessions had lower total psychotic symptoms after treatment than those who received fewer sessions (Turner et al., 2020). The NICE guidelines in the United Kingdom recommend a minimum of 16 CBTp sessions, and the Dutch psychosis care guidelines suggest a range of 16–35 sessions (Haddock et al., 2014). In clinical practice, there seems to be a considerable variation in the number of sessions delivering CBTp, which may be caused by a lack of clear evidence and the heterogeneity in the problems and goals of patients, but is also explained by variations in human and financial resources (Burgess-Barr et al., 2023). Given the limited availability of trained psychologists in mental healthcare, increasing treatment efficiency is crucial for improving access to psychological treatment for patients with a psychotic disorder.

We conducted a randomized clinical trial comparing VR-CBTp for paranoid ideation to standard CBTp (Berkhof et al., 2021). The main aim was to investigate differences between VR-CBTp and CBTp in their effect on the level of paranoid ideation in daily life, the level of social activities, the proportion of time spent in social company, levels of distress, anxiety, and depression. In addition, we aimed to determine whether the number of required treatment sessions differs between CBTp and VR-CBTp. We hypothesized that VR-CBTp would show greater symptom reduction (i.e. paranoia in daily life) and larger effects on secondary outcomes (e.g. distress, depression, anxiety, self-esteem, and safety behavior). Furthermore, we expected VR-CBTp to reach these effects in fewer treatment sessions than CBTp.

## Methods

### Study design

We performed a pragmatic, single-blind, multicenter, randomized clinical superiority trial comparing two arms: VR-CBTp and CBTp (Berkhof et al., 2021). Participants were outpatients recruited from six mental healthcare centers in the Netherlands and one in Belgium. The centers were dispersed over the Netherlands and Belgium, ensuring the recruitment of people from diverse environments, including rural, urban, and suburban environments. Therapists were employed at these centers and delivered the treatments in the trial as part of their regular clinical work. The trial was approved by the medical ethical committee of the University Medical Centre Groningen (number NL66850.042.18), and prospectively registered in the Dutch trial register (migrated to the Central Committee on Research Involving Human Subjects [CCMO] register) with trial number N7758.

### Participants

Inclusion criteria were a *Diagnostic and Statistical Manual of Mental Disorders*, Fifth Edition, diagnosis of a schizophrenia spectrum or other psychotic disorder, age 18–65 years, having an indication for treatment by a clinician, and paranoid ideation verified by a minimum score of 40 in the Green et al. Paranoid Thoughts Scale (GPTS) (Green et al., 2008). Exclusion criteria were an estimated IQ below 70, insufficient command of the Dutch language, and having received CBTp for paranoid ideation in the past 12 months.

### Procedures

Patients were recruited through advertisements (posters and flyers) at the participating centers and through clinicians who informed and referred patients. If a patient was willing to participate, written informed consent was obtained, after which the GPTS was completed to check eligibility (GPTS score > 40).

Participants were randomly allocated after the baseline assessment. Block randomization was used, with blocks of eight assignments generated by www.randomizer.org for each center. Randomization was carried out by an independent coordinator of the University Medical Center Groningen. Participants could receive additional types of treatment as usual during the study period, including antipsychotic medication, except for any form of CBT.

Participants were assessed at baseline (t0), posttreatment within 2 weeks after completing treatment (t1), and at the 3-month follow-up (t2). Assessors were blinded to treatment allocation and had to be replaced in case of unblinding. Blinding was evaluated with a self-report form. In 12 out of 160 posttreatment and follow-up assessments, assessors indicated that they were unblinded (2 indicated the wrong group). Two assessors were replaced; one participant only wanted to be assessed by the unblinded assessor, and in nine cases, replacement was undesirable due to increased burden when an additional assessment had to be planned. Sensitivity analyses were performed, testing effects for interview outcomes where assessors were completely unblinded (see Supplement 1).

### VR-CBTp and CBTp interventions

Both VR-CBTp and CBTp consisted of a maximum of 16 individual sessions delivered within 8–12 weeks, and sessions lasted a maximum of 75 min. For CBTp, the Dutch CBT protocol for paranoid ideation was used (www.gedachtenuitpluizen.nl). The VR-CBTp protocol was based on this protocol as well. The CBTp protocol starts with constructing individual case formulations in collaboration with the patient to create an understanding of the paranoid ideas, and related feelings and behavior (in the first two sessions). Further, the protocol consists of cognitive techniques, such as cognitive restructuring, and behavior interventions, including exposure and behavior experiments. It focuses strongly on exercises (and planning and reflection thereof) aimed at the reappraisal of paranoid beliefs to reduce distress and improve the coping of patients.

Therapy could be completed before 16 sessions in case goal behaviors were achieved, all parts of the safety circle were effectively treated, and participants scored 0 on the Subjective-Units-of-Distress scale for identified fear cues in two consecutive sessions. In addition, session measurements should show a decrease in paranoid ideation, distress, and avoidance. Before stopping, the case had to be discussed with a supervisor.

The main difference between the two protocols was that VR-CBTp exercises were performed in VR during sessions, for ±40 min per session (from session 3 onwards), using social situations that triggered paranoid ideation and distress. In VR, patients were guided by therapists to drop safety behaviors, test paranoid beliefs, and engage in new behavior. Exercises could be tailored to the participant’s needs and goals and could be repeated. In contrast, in vivo exposure and behavior exercises during CBTp started after session 7.

For VR-CBTp, the CE-marked Social Worlds software (CleVR, Delft, The Netherlands) was used to role-play personalized and interactive scenarios. The VR was offered with an Oculus Rift head-mounted display (Reality Labs, Menlo Park, USA), and patients navigated through VR environments using a controller. Multiple VR environments were used: a café, a shopping street, a supermarket, a bus ride, an office, and a living room. The difficulty level could be modified by adjusting the number, gender, gestures, and ethnic appearance of virtual characters (avatars) present in the VR social situations. Moreover, the level of hostility and suspicious behavior could be modified. During scenarios, therapists used a microphone with voice distortion to be able to speak as an avatar.

Both VR-CBTp and CBTp were delivered by psychologists with at least a postmaster qualification in CBT (100 h of accredited CBT training) and a minimum of 6 months of experience in psychosis treatment. Therapists were trained in both protocols during a 4-day training and supervised by experienced psychologists registered at the Dutch Association of Behaviour and Cognitive Therapies (VGCt). Individual case conceptualizations were guided and evaluated by a supervisor after session 2. Therapists participated in monthly 2-h group supervision sessions. Treatments were audio recorded, and two recordings per therapist were rated for treatment fidelity by author MH (psychologist) using the Cognitive Therapy Rating Scale. Both the session number and participant were assigned randomly to ensure unbiased selection for rating.

### Outcomes

The primary outcome was momentary paranoia in daily life at posttreatment, measured with the experience sampling method (ESM). ESM is a structured diary method; participants completed diary questionnaires on their mobile phones at semi-random moments 10 times a day for 7 days. ESM affect items were scored on visual analogue scales (VAS) ranging from 0 (*not at all*) to 100 (*very*). Momentary paranoia was assessed by the following items: ‘I feel that others might hurt me’, ‘I feel that others dislike me’, and ‘I feel suspicious’. A minimum of 30% of the questionnaires had to be completed for analyses. Mean total scores were analyzed. We performed a principal component analysis with oblique rotation and Kaiser normalization on the person-centered items to confirm subscales from the literature. The negative effect, positive effect, and three-item momentary paranoia subscale (factor loadings of momentary paranoia ranged from 0.63 to 0.91) were confirmed.

Secondary ESM outcomes concerned the proportion of time in social company (‘With whom have I been since the previous measurement?’) and social activities, in terms of social threat both in the current moment and since the previous measurement. Social threat was assessed using the mean of the following items: (i) ‘I like this company [reversed score]’, (ii) ‘In this company, I feel accepted [reversed score]’, and (iii) ‘In this company, I feel unsafe’). To avoid/endure threat situations, the mean of the following items was assessed: (i) ‘Since the previous measurement, I have avoided situation or activities to avoid danger/fear’ and (ii) ‘Since the previous measurement, I have had difficulty coping with situations or activities that I could not avoid’).

Secondary interview outcomes were the Psychotic Symptom Rating Scales (PSYRATS) (Haddock, McCarron, Tarrier, & Faragher, 1999) and the Safety Behaviours Questionnaire–persecutory delusions (Freeman, Garety, & Kuipers, 2001). Self-report questionnaire outcomes were the GPTS (additionally, R-GPTS scores were calculated) (Freeman, Loe, et al., 2021), Social Interaction Anxiety Scale (Mattick & Clarke, 1998), Inventory of Depressive Symptomatology Self-Report (Rush et al., 1996), Self-Esteem Rating Scale–Short Form (Lecomte, Corbière, & Laisné, 2006), Interpersonal Sensitivity Measure (Boyce & Parker, 1989), Brief Core Schema Scales (Fowler et al., 2006), Davos Assessment of Cognitive Biases (van der Gaag et al., 2013), social functioning (PSP) (Kawata & Revicki, 2008), and Penn State Worry Questionnaire (Brown, Antony, & Barlow, 1992).

### Statistical analyses

A previous RCT comparing VR-CBTp with waiting list reported a large effect size (ES) (*d* = 1.6) for our primary outcome, that is, momentary paranoia (Pot-Kolder et al., 2018). The ES was expected to be smaller with standard CBTp as a comparison; therefore, we aimed to detect an ES of 0.6. Assuming a power of 0.8, *α* of 0.05, and a standard deviation (SD) of 1.1, the sample size was calculated to be 106 to detect an ES of 0.6 between groups. While allowing a 15% attrition rate, the total sample size was calculated to be 122.

Data were analyzed by intention to treat with SPSS (version 29.0.1.0). A *p*-value < .05 was considered significant. The Mann–Whitney *U* test was done to analyze differences in the number of sessions. Multilevel regression analyses with a random intercept for participants, identity covariance structure, and maximum likelihood method for estimation were performed. The treatment effect was established with the group-by-time interaction for posttreatment and follow-up separately, as well as the main effects of group and time. Cohen’s *d* effect sizes were calculated (by dividing the estimated mean difference by the pooled SD) for the primary outcome and related paranoia measures (PSYRATS and GPTS).

## Results

**Figure 1.**
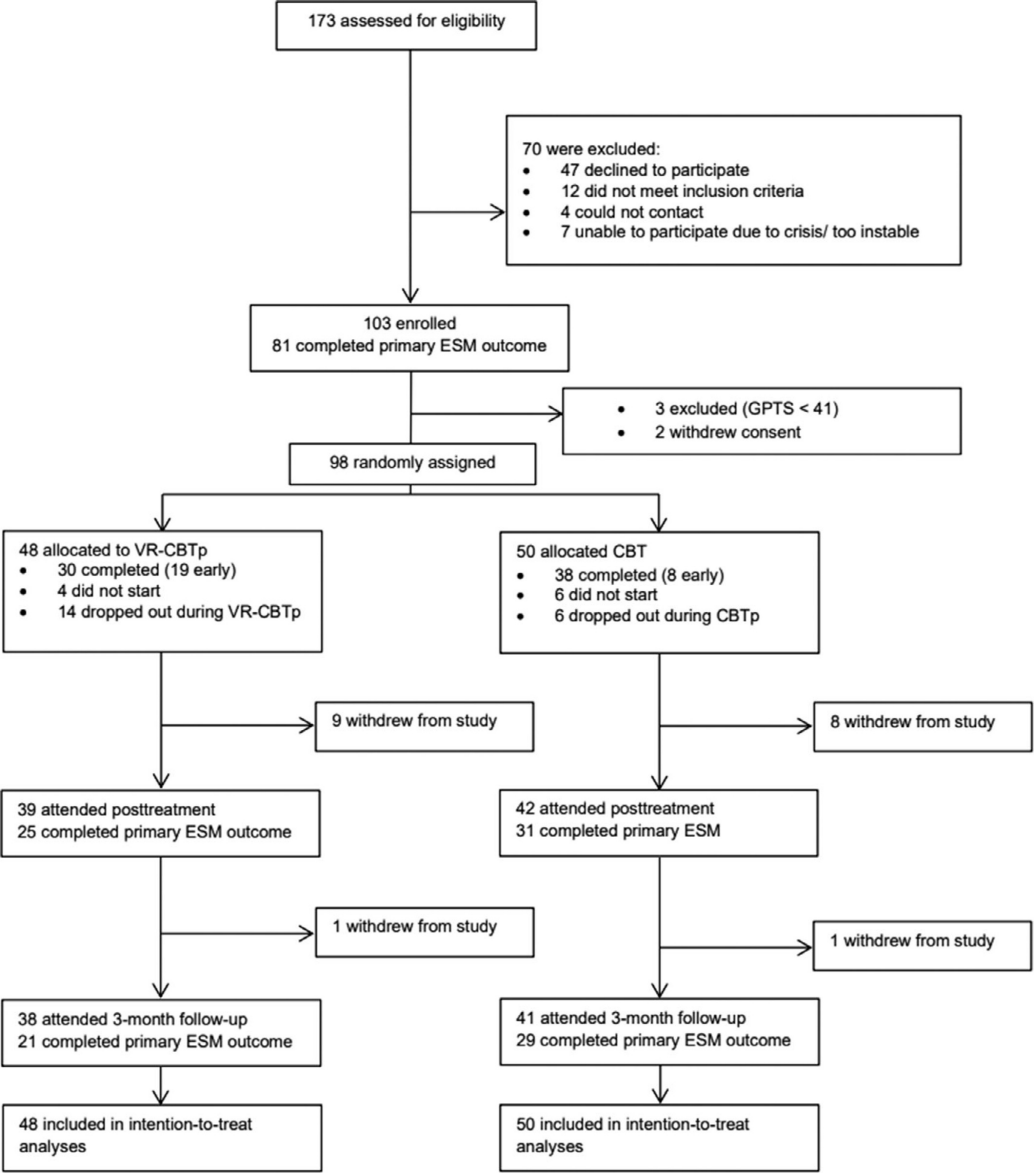
Flowchart of the trial.

A total of 103 patients were enrolled between October 1, 2019, and July 31, 2023 (Figure 1). Three participants were excluded because of a GPTS < 40, and two withdrew consent before randomization. In total, 98 were randomized to VR-CBTp (*n* = 48) or CBTp (*n* = 50). Of the randomized patients, 27% were female, 73% were male, and the mean age was 35.8 years (SD = 2.6) (Table 1). Baseline characteristics were well-balanced between groups, although schizophrenia was more common in the VR-CBTp group.

**Table 1.** Baseline characteristics

	VR-CBTp *n* = 48	CBTp *n* = 50
Age, years	35.5 (12.9)	36.1 (12.3)
Sex		
Male	33 (68.8%)	39 (78.0%)
Female	15 (31.3%)	11 (22.0%)
Dutch origin	40 (83.3%)	39 (78.0%)
Education		
None or primary	4 (8.3%)	2 (4.0%)
Secondary	22 (46.1%)	23 (46.0%)
Vocational	14 (29.2%)	18 (36.0%)
Higher	8 (16.9%)	7 (14.0%)
Employment status		
Student	8 (16.7%)	6 (12.0%)
Employed full-time	4 (8.3%)	3 (6.0%)
Employed part-time	8 (16.5%)	7 (14.0%)
Unemployed	6 (12.5%)	15 (30.0%)
Unfit for work	16 (33.3%)	13 (26.0%)
Volunteer work	4 (8.3%)	2 (4.0%)
Day activity care	0	2 (4.0%)
Other	2 (4.2%)	2 (4.0%)
DSM–5 psychosis spectrum diagnosis		
Unspecified schizophrenia spectrum and other psychotic disorder	18 (37.5%)	23 (46.0%)
Schizophrenia	14 (29.2%)	7 (14.0%)
Schizoaffective disorder	5 (10.4%)	5 (10.0%)
Delusional disorder	6 (12.5%)	5 (10.0%)
Other specified schizophrenia spectra and other psychotic disorder	2 (4.2%)	6 (12.0%)
Brief psychotic disorder	1 (2.1%)	4 (8.0%)
Schizophreniform disorder	1 (2.1%)	0
Schizotypal personality disorder	1 (2.1%)	0
Prescribed an antipsychotic medication	43 (89.6%)	39 (78.0%)
Prescribed an antidepressant medication	15 (31.3%)	13 (26.0%)

*Note:* Data are *n* (%) or mean (standard deviation).

A total of 18 participants (37.5%) dropped out of VR-CBTp and 12 (24.0%) of CBTp (Table 2). Of those, four in the VR-CBTp group never started therapy because of symptom reduction (*n* = 2), unwillingness (*n* = 1), and other treatment required (*n* = 1). Reasons for dropout during VR-CBTp were other treatment required (*n* = 3) lost contact/unknown (*n* = 3), treatment too intensive (*n* = 3), unwillingness (*n* = 2), assessment too intensive (*n* = 1), severe physical illness ( = 1), and exacerbated symptoms/no trust in care (*n* = 1). CBTp participants dropped out before the start of treatment due to imprisonment (*n* = 1), other treatment required (*n* = 2), assessment too intensive (*n* = 1), treatment too intensive (*n* = 1), and exacerbated symptoms (*n* = 1). During CBTp, people dropped out because of exacerbated symptoms/no trust in care (*n* = 3), other treatment required (*n* = 1), unwillingness (*n* = 1), and lost contact (*n* = 1).

**Table 2.** Treatment characteristics

	VR-CBTp *n* = 48	CBTp *n* = 50
Number of sessions	9.4 (5.6)	12.3 (6.0)
**Treatment completers**	***n* = 30 (62.5%)**	***n* = 38 (76.0%)**
Number of sessions mean	12.7 (3.9)	15.1 (2.5)
Number of sessions		
1–4 sessions	1 (3.3%)	0
5–8 sessions	4 (13.3%)	2 (5.3%)
9–12 sessions	7 (23.3%)	1 (2.6%)
13–16 sessions	18 (60.0%)	35 (92.1%)
**Treatment dropouts**	***n* = 18 (37.5%)**	***n* = 12 (24.0%)**
Number of sessions		
0 sessions	4 (22.2%)	6 (50.0%)
1–4 sessions	5 (27.8%)	2 (16.7%)
5–8 sessions	9 (50.0%)	1 (8.3%)
9–12 sessions	0	2 (16.7%)
13–16 sessions	0	1 (8.3%)

*Note:* Data are *n* (%) or mean (standard deviation).

Treatment dropout was higher during the coronavirus disease 2019 (COVID-19) restriction period (March 2020–March 2022) for VR-CBTp (40.0% vs. 34.8%) and CBTp (25.9% vs. 21.7%). Participants who dropped out did not have higher baseline paranoia (PSYRATS and GPTS) than completers. When considering only treatment completers, VR-CBTp took 12.7 (SD 3.9) sessions and CBTp took 15.1 sessions (SD = 2.5, *U* = 738.5, *p* < .001). Of the completers, 56.7% of the VR-CBTp group were early completers, completing treatment before the 16th session, and for CBTp, this was 21.1%.

In total, 24 therapists participated in the trial, of whom 4 had no recordings. Of the 1,036 sessions, over three-fifths had recordings. A total of 40 sessions (2 per therapist) were rated for treatment fidelity and showed good to very good adherence in both groups (*M*_CBT_ = 4.7, SD = 0.6 and *M*_VR-CBGTp_ = 4.8, SD = 0.7) on a 6-point scale. The session with the lowest adherence scored 3.5, and the highest session score was 5.7.

At baseline, 81 of 98 randomized participants sufficiently completed ESM, with a mean of 47.9 self-assessments (SD = 13.9). At posttreatment, 56 participants (57% of all randomized participants and 69% of those with baseline data) completed ESM (*M* = 44.4, SD = 12.7). At follow-up, 50 participants (51% of randomized participants and 62% of those with baseline data) completed the ESM (*M* = 42.5, SD = 12.5).

Momentary paranoia (primary outcome) improved significantly between baseline and posttreatment in both groups (ES = 0.65), as well as between baseline and follow-up (ES = 0.57) (see Tables 3 and 4). Participants in the VR-CBTp group improved on average by 12.6 points and the CBTp group by 9.2 points between baseline and follow-up. A significant interaction effect in favor of VR-CBTp was found at posttreatment (ES = 0.62) but not at follow-up (ES = 0.31).

**Table 3.** Means and standard deviations of outcomes over time

		VR-CBTp			CBTp		
		Baseline	Post	Follow-up	Baseline	Post	Follow-up
**Primary outcome**		***n* = 40**	***n* = 25**	***n* = 21**	***n* = 41**	***n* = 31**	***n* = 29**
Momentary paranoia	ESM	27.7 (17.7)	12.7 (11.6)	15.1 (11.6)	27.5 (22.4)	18.3 (22.4)	18.3 (19.6)
**Interviews**		***n* = 48**	***n* = 38**	***n* = 37**	***n* = 50**	***n* = 42**	***n* = 41**
Paranoid delusions	PSYRATS	13.3 (5.9)a	7.7 (6.7)	6.6 (7.3)b	12.2 (6.4)	9.1 (7.3)	8.8 (7.1)
Verbal hallucinations	PSYRATS	7.1 (10.2)	5.4 (9.1)	3.6 (7.9)	5.0 (9.5)	2.7 (6.8)	2.7 (7.1)c
Safety behaviors – avoidance	SBQ-PD	7.3 (7.4)	2.7 (4.7)	3.2 (6.2)	5.4 (5.1)	4.3 (5.9)	3.8 (5.4)
Safety behaviors – in situation	SBQ-PD	5.3 (6.2)d	2.3 (2.9)e	2.0 (3.5)e	3.8 (3.9)f	2.7 (3.5)g	1.6 (2.9)h
**Self-report questionnaires**		***n* = 48**	***n* = 39**	***n* = 38**	***n* = 50**	***n* = 42**	***n* = 40**
Paranoia –ideas of social reference	GPTS	45.8 (14.0)	29.2 (12.0)	31.3 (14.1)	47.7 (12.9)	33.0 (15.1)	34.1 (14.9)
Paranoia – ideas of persecution	GPTS	43.8 (14.6)	28.4 (13.7)	30.1 (15.5)	47.8 (16.4)	31.0 (17.7)	34.3 (18.5)
Ideas of social reference	R-GPTS	14.4 (8.0)	6.6 (6.1)	7.4 (6.6)	14.7 (6.9)	8.4 (7.2)	8.5 (7.3)
Ideas of persecution	R-GPTS	17.2 (9.5)	7.7 (8.8)	9.1 (9.9)	20.0 (10.3)	9.4 (11.1)	11.4 (11.4)
Social interaction anxiety	SIAS	32.6 (16.4)	24.7 (13.4)	22.9 (13.9)	33.7 (16.6)	30.4 (14.5)	30.4 (15.3)
Depression symptoms	IDS-SR	28.4 (15.7)	20.3 (12.7)	20.8 (15.2)	27.0 (13.2)	24.9 (14.9)	21.8 (14.4)
Worry	PSWQ	53.7 (12.7)	45.8 (14.8)	46.3 (14.0)	54.3 (12.5)	50.7 (13.8)	47.5 (14.3)
Cognitive problems and biases	DACOBS	171.3 (32.1)	149.4 (30.6)	149.9 (41.4)	171.3 (30.2)	160.0 (39.9)	159.8 (35.8)
Interpersonal sensitivity	IPSM	58.0 (18.1)i	55.6 (15.5)j	54.8 (20.6)	60.6 (18.9)k	58.6 (16.6)	58.5 (18.8)
Social functioning	PSP	48.9 (12.2)	58.9 (16.2)	58.6 (17.0)	47.7 (15.8)	52.7 (18.1)	51.0 (16.7)
Positive self-esteem	SERS	40.7 (11.8)	45.3 (12.3)	46.6 (13.5)	40.6 (11.9)	40.0 (10.7)	40.7 (12.6)
Negative self-esteem	SERS	37.9 (14.8)	29.4 (14.1)	28.2 (13.9)	34.6 (13.5)	32.3 (13.5)	32.7 (14.0)
Negative self-core schema	BCSS	5.5 (5.5)	3.5 (4.3)	3.2 (4.1)	5.0 (4.5)	4.0 (4.1)	4.0 (4.3)
Positive self-core schema	BCSS	8.3 (5.5)	10.7 (6.1)	11.2 (7.0)	8.9 (5.2)	9.6 (5.4)	9.6 (5.7)
Negative others’ core schema	BCSS	6.8 (5.7)	4.2 (4.9)	6.0 (6.0)	6.8 (5.8)	6.0 (6.3)	4.6 (5.2)
Positive others’ core schema	BCSS	7.1 (5.2)	8.3 (6.1)	8.5 (6.5)	9.0 (5.2)	8.7 (5.5)	9.5 (5.9)
**ESM**		***n* = 40**	***n* = 25**	***n* = 21**	***n* = 41**	***n* = 31**	***n* = 29**
Avoid/endure threat situations	ESM	23.0 (16.5)	11.4 (11.1)	12.2 (12.6)	20.3 (15.8)	11.4 (12.0)	11.2 (11.5)
Proportion of time in company	ESM	0.61 (0.29)	0.61 (0.24)	0.58 (0.29)	0.51 (0.27)	0.54 (0.28)	0.52 (0.25)
Social threat (now)	ESM	25.3 (12.4)	20.2 (12.8)	23.8 (13.7)	19.4 (13.3)	19.1 (13.8)	20.2 (14.3)
Social threat (since previous diary)	ESM	28.1 (13.0)	22.2 (12.4)	25.6 (14.5)	20.9 (13.1)	20.9 (13.0)	20.5 (13.5)

*Note:* Data are mean (SD). Deviating *n*: ^a^= 47, ^b^= 36, ^c^ = 40, ^d^= 43, ^e^= 26, ^f^= 42, ^g^= 35, ^h^= 34, ^i^ = 45, ^j^ = 38, ^k^ = 48.

**Table 4.** Test results (main and interaction effects) of multilevel analyses

	Posttreatment							Follow-up				
	Main effect of time		Main effect group		Interaction effect		Main effect of time		Main effect group		Interaction effect	
	*b* (95% CI)	*p*	*b* (95% CI)	*p*	*b* (95% CI)	*p*	*b* (95% CI)	*p*	*b* (95% CI)	*p*	*b* (95% CI)	*p*
**Primary outcome**												
Momentary paranoia	−15.0 (−21.0: −9.0)	**<.001**	−0.3 (−8.4: 7.8)	.94	8.3 (0.2: 16.4)	**.044**	−10.7 (−16.8: −4.6)	**<.001**	−0.5 (−9.0: 8.0)	.90	4.1 (−4.0: 12.2)	.31
**Interviews**												
Paranoid delusions	−5.5 (−8.0: −3.0)	**<.001**	−1.1 (−3.7: 1.5)	.39	2.3 (−1.1: 5.8)	.19	−6.6 (−8.9: −4.2)	**<.001**	−1.1 (−3.7: −1.5)	.41	3.0 (−0.2: 6.3)	.07
Verbal hallucinations	−2.6 (−6.1: 1.0)	**.023**	−2.8 (−7.6: 2.1)	.26	0.6 (−5.4: 4.3)	.82	−4.8 (−8.3: −1.4)	**<.001**	−2.8 (−7.3: 1.9)	.24	1.5 (−3.3: 6.3)	.52
Safety behaviors – avoidance	−4.9 (−6.9: −2.9)	**<.001**	−1.9 (−4.2: 0.4)	.11	3.7 (0.9: 6.5)	**.009**	−4.4 (−6.4: −2.4)	**<.001**	−1.9 (−4.3: 0.5)	.12	2.7 (−0.-: 5.5)	.054
Safety behaviors – in situation	−3.4 (−5.2: −1.5)	**.003**	−1.9 (−3.8: 0.0)	.054	2.8 (0.3: 5.3)	**.028**	−3.5 (−5.2: −1.8)	**<.001**	−1.8 (−3.7: 0.1)	.06	1.8 (−0.5: 4.2)	.12
**Self-report questionnaires**												
Paranoia – ideas of social reference	−16.9 (−21.4: −12.4)	**<.001**	1.9 (−3.5: 7.3)	.48	2.2 (−4.1: 8.4)	.49	−14.4 (−19.2: 9.5)	**<.001**	1.9 (−3.6: 7.4)	.50	0.6 (−6.1: 7.4)	.85
Paranoia – ideas of persecution	−15.9 (−20.7: −11.0)	**<.001**	4.0 (−2.1: 10.2)	.20	−0.7 (−7.5: 6.0)	.83	−13.8 (−18.8: −8.9)	**<.001**	4.0 (−2.4: 10.4)	.21	0.2 (−6.8: 7.1)	.96
Ideas of social reference	−8.0 (−10.4: −5.5)	**<.001**	0.3 (−2.5: 3.1)	.82	1.5 (−1.9: 4.9)	.38	−7.0 (−9.4: −4.4)	**<.001**	0.3 (−2.6: 3.2)	.82	0.7 (−2.9: 4.3)	.70
Ideas of persecution	−9.9 (−12.9: −6.8)	**<.001**	2.8 (−1.2: 6.7)	.17	−0.7 (−4.9: 3.6)	.76	−8.3 (−11.4: −5.3)	**<.001**	2.8 (−1.3: 6.8)	.18	−0.4 (−4.7: 3.9)	.84
Social interaction anxiety	−9.1 (−12.9: −5.2)	**<.001**	1.1 (−5.1: 7.2)	.73	4.2 (−1.1: 9.6)	.12	−11.0 (−15.1: −7.0)	**<.001**	1.1 (−5.2: 7.3)	.73	6.3 (0.7: 12.0)	**.029**
Depression symptoms	−9.5 (−13.1: −5.9)	**<.001**	−1.4 (−7.1: 4.2)	.61	7.2 (2.2: 12.2)	**.005**	−8.7 (−12.7: −4.8)	**<.001**	−1.4 (−7.2: 4.3)	.62	3.4 (−2.1: 8.9)	.23
Worry	−0.8 (−11.6: −4.5)	**<.001**	0.6 (−4.7: 5.9)	.82	3.7 (−1.2: 8.7)	.14	−7.2 (−10.6: −3.8)	**<.001**	0.6 (−4.7: 5.9)	.82	0.1 (−4.7: 4.8)	.98
Cognitive problems and biases	−22.3 (−33.0: −11.5)	**<.001**	0.0 (−13.1: 13.2)	.99	11.3 (−3.6: 26.2)	.14	−21.8 (−33.3: −10.4)	**<.001**	0.0 (−13.6: 13.7)	.99	10.5 (−5.4: 26.5)	.19
Social functioning	9.6 (4.7: 14.3)	**<.001**	−1.2 (−7.4: 4.9)	.69	−4.9 (−11.5: 1.8)	.15	9.3 (4.5: 14.0)	**<.001**	−1.2 (−7.3)	.69	−6.1 (−12.8: 0.4)	.07
Interpersonal sensitivity	−4.5 (−9.0: 0.1)	**.014**	0.7 (−6.5: 7.9)	.86	1.1 (−5.2: 7.3)	.73	−4.2 (−9.9: 1.5)	.08	0.8 (−6.9: 8.4)	.84	1.4 (−6.6: 9.3)	.74
Positive self-esteem	4.0 (1.4: 6.6)	**.045**	−0.1 (−4.7: 4.6)	.97	−4.3 (−8.0: −0.7)	**.019**	5.4 (2.6: 8.2)	**<.001**	−0.1 (−5.0: 4.8)	.97	−5.1 (−9.0: −1.2)	**.011**
Negative self-esteem	5.0 (0.1: 9.8)	**<.001**	−3.2 (−8.8: 2.3)	.25	5.0 (0.1: 9.8)	**.044**	−9.2 (−12.6: −5.8)	**<.001**	−3.2 (−8.8: 2.4)	.26	6.0 (1.3: 10.7)	**.013**
Negative self-core schema	−1.8 (−3.2: 0.5)	**<.001**	– 0.5 (−2.4: 1.3)	.58	0.4 (−1.4: 2.3)	.63	−2.0 (−3.2: −0.7)	**<.001**	−0.5 (−2.4: 1.3)	.58	0.5 (−1.2: 2.2)	.58
Positive self-core schema	1.2 (−.4: 1.9)	**.007**	0.5 (−1.8: 2.7)	.68	−0.9 (−1.9: 0.2)	.10	0.4 (0.0: 0.7)	.053	0.1 (−2.1: 2.2)	.95	−0.2 (−0.7: 0.3)	.36
Negative others’ core schema	−2.6 (−4.1: −1.0)	**.003**	−0.0 (−2.3: 2.2)	.98	1.9 (−0.2: 4.0)	.07	−0.8 (−2.3: 0.7)	**.008**	0.0 (−2.3: 2.2)	.98	−1.3 (−3.4: 0.8)	.24
Positive others’ core schema	1.2 (−0.1: 2.5)	.35	1.9 (−0.3: 4.0)	.09	−1.6 (−3.4: 0.2)	.08	1.3 (−0.3: 2.9)	.16	1.9 (−0.4: 4.1)	.10	−0.9 (−3.2: 1.3)	.41
**ESM**												
Avoid/endure threat situations	−5.8 (−10.4: −1.2)	**<.001**	2.7 (−3.8: 9.2)	.41	−6.0 (−12.8: 0.8)	.08	−3.0 (−5.2: −0.9)	**<.001**	2.9 (−3.8: 9.6)	.39	−1.3 (4.5: 1.9)	.42
Proportion time in company	−0.04 (−0.13: 0.05)	.59	−0.10 (−0.22: 0.02)	.09	0.04 (−0.08: 0.16)	.50	−0.05 (−0.16: 0.05)	.43	−0.11 (−0.23: 0.01)	.08	0.05 (−0.09: 0.19)	.45
Social threat (now)	−4.8 (−8.9: −0.8)	.08	−5.4 (−11.1: 0.2)	.06	4.8 (−0.7: 10.3)	.09	−1.3 (−4.7: 2.1)	.84	−5.9 (−11.7: −0.17)	**.04**	3.0 (−1.4: 7.50)	.18
Social threat (since previous diary)	−3.5 (−6.9: −0.2)	.11	−6.7 (−12.4: −1.0)	**.02**	3.4 (−1.1: 7.9)	.13	−1.1 (−4.0: 1.8)	.48	−7.1 (−12.9: −1.4)	**.02**	0.9 (−2.9: 4.7)	.65

*Note*: CBTp was coded as 0 and VR-CBTp as 1. *p*-values derived from Type III F tests of fixed effects, confidence intervals from estimates of *t* fixed effects.

The PSYRATS interview confirmed the change in paranoid delusions; both groups reported reductions in paranoid delusions over time, with the biggest change occurring between baseline and posttreatment (ES_t0–t1_ = 0.66 and ES_t0–t2_ = 0.74). No significant interaction effects were found for posttreatment (ES = 0.17) nor follow-up (ES = 0.32); thus, the two therapies did not produce significantly different outcomes on these paranoia measurements. Similarly, the GPTS showed that patients in both treatment groups showed reduced ideas of social reference (ES_t0–t1_ = 1.15 and ES_t0–t2_ = 1.01) and ideas of persecution (ES_t0–t1_ = 1.02 and ES_t0–t2_ = 0.83) with strong improvements at the post-assessment. No significant interaction effects for ideas of social reference (ES_t0–t1_ = 0.17 and ES_t0–t2_ = 0.18) and persecution were found (ES_t0–t1_ = 0.00 and ES_t0–t2_ = 0.01).

Participants showed significantly less safety behavior over time. This effect was stronger in the VR-CBTp group, as shown by the significant interaction effect at posttreatment for avoidance and in-situation safety behavior, but this effect did not reach significance at follow-up. With regard to social interaction anxiety, patients in both treatment arms showed reduced social interaction anxiety post-treatment (main effects of time significant at *p* < .001 for both time points). There was no significant interaction effect at posttreatment, but we did observe stronger improvements in the VR-CBTp group at follow-up. In addition, symptoms of depression, worry, cognitive problems and biases, and social functioning showed improvements over time when looking at both groups (*p* < .001). Only for depression, an interaction effect was found, reflecting stronger improvement in the VR-CBTp group at posttreatment but not at follow-up. Interpersonal sensitivity also improved slightly in both groups, but this was only significant for posttreatment.

For self-esteem and core schemas, significant main effects of time were found for posttreatment and follow-up, except regarding positive others’ core schemas. For positive and negative self-esteem, significant interaction effects were established for both time points, reflecting that self-esteem improved over time in the VR-CBTp group but remained stable over time in the CBTp group.

Regarding secondary ESM outcomes, a significant improvement was found for the avoid/endure threat situations scale over time between baseline and posttreatment, as well as follow-up. No significant improvements over time were found for proportion in time in the company or social threat subscales. There was a main effect of group for the social threat scales that was attributable to the difference at baseline.

A total of 15 serious adverse events occurred during the trial (*n* = 2 before randomization; *n* = 3 VR-CBTp; *n* = 10 CBTp). Two serious events (admissions) were possibly related to the trial, specifically to the intensity of the baseline assessment and more awareness of behavior patterns that led to an increase in suffering after the first session.

## Discussion

In this RCT comparing VR-CBTp to standard CBTp for paranoid ideation in patients with a psychotic disorder, we found that following both treatments, patients reported significantly reduced momentary paranoia and severity of paranoid delusions, safety behavior, social anxiety, depression, worry, and dysfunctional cognitive schemes. Furthermore, posttreatment, patients reported improved self-esteem and social functioning, and most effects were maintained 3 months after treatment. At posttreatment, VR-CBTp had significantly stronger effects than standard CBTp on momentary paranoia, safety behavior, depressive symptoms, and self-esteem, of which the difference in effects on self-esteem and social anxiety remained at follow-up. In VR-CBTp, 37% of the participants dropped out versus 24% in the CBTp group. Completers, on average, received 12.7 (VR-CBTp) and 15.1 (CBTp) sessions. These findings indicate that CBTp and VR-CBTp are both efficacious treatments for paranoid ideation but that VR-CBTp may be somewhat more effective directly after treatment completion.

Treatment dropout rates were substantially higher in the VR-CBTp group (37%) than in the CBTp group (24%). A substantial proportion of the dropouts (22% and 50%, respectively) did not start therapy. Reasons for dropout were often unrelated to the interventions (prioritizing other treatment, e.g. for trauma, assessment too intensive, imprisonment, and physical illness). With regard to VR-CBTp, sometimes participants had to travel further than usual, as VR sets were located at specific locations, which may have influenced willingness to participate. Moreover, high dropout could be partly caused by the COVID-19 pandemic, as outside the pandemic, dropout was lower. It may also be attributed to the rapid emphasis on exposure in VR-CBTp, which might be less tolerable for some, or to a perceived mismatch between the possibilities for behavior exercises in VR and the goals of participants. The strong focus on exposure/behavior exercises might make the treatment less tolerable or suitable for some, but it is also a major strength of VR-CBTp. Lastly, dropout may be attributed to the pragmatic design in which therapy was integrated into a clinical setting. However, this design enabled a realistic image of feasibility in clinical practice.

To improve engagement and reduce dropout, hybrid treatments – combining VR with in vivo exercises and telehealth options where patients can be partly treated at home – could form a solution. Also, more precise treatment indication (as dropout was often related to a mismatch with the patient’s current needs), combined with shared decision-making regarding treatment choices, could enhance the effectiveness and acceptability of the interventions. Finally, portable VR sets or VR sets at more treatment locations are necessary to counter patients’ traveling.

When we look at those participants who completed therapy, we observe that similar treatment effects were attained in VR-CBTp with fewer sessions. The VR-CBTp protocol explicitly includes extensive practice in VR, stimulating patients and therapists to engage in exposure for 40 min per session, whereas research has shown that in vivo exposure is frequently underutilized (de Jong et al., 2020). This enabled participants to practice in threatening social situations earlier, more often, and more intensively and in a personalized way. The working mechanism of behavior exercises is dropping safety behaviors to experience high levels of anxiety and to subsequently learn that one is safe and can endure distress; that is, threat beliefs are falsified, and maladaptive fear associations may be replaced with new, more adaptive ones. This approach may thus facilitate quicker and stronger reduction of safety behavior than in CBTp. Consequently, it could have increased the experience of positive learning effects and impacted self-esteem. Possibly, the direct VR exposures are such an empowering experience that improves self-esteem.

This is consistent with current cognitive behavior models of paranoid delusions that view delusions as threat beliefs maintained by the use of safety strategies and psychological factors such as worry and low self-esteem (Freeman, 2016). If treatment successfully targets these maintaining factors, (Freeman, Emsley, et al., 2021), patients will more easily enter feared situations and will experience that they are safe, as a result of which paranoid ideation will gradually disappear. VR-CBTp centers around behavior exercises and lowers the threshold for engaging in threatening virtual social situations because patients know it is not real, virtual scenarios can be completely controlled, and the therapist is present for guidance. Indeed, results from our previous VR-CBTp RCT confirmed that the reduction in safety behavior over time was related to diminished levels of paranoid ideation (Pot-Kolder et al., 2018).

This finding of potentially higher treatment efficiency of VR-CBTp is important both for patients and mental healthcare, especially when replicated. While both conditions used similar symptom-oriented protocols focusing on paranoid ideation, we cannot rule out the possibility that VR-CBTp may have had a somewhat narrower focus than CBTp. Nonetheless, as results indicated that there was generalization to real-life situations for both treatments with similar effects on a wide range of outcomes, they imply higher efficacy of the VR-CBTp protocol. Quicker recovery is advantageous for people suffering from paranoid delusions, as these symptoms have been associated with high levels of distress and hospital admissions (Freeman et al., 2014). A 16% reduction in treatment duration (2.6 of 16 sessions) with VR-CBTp compared to standard CBTp is relevant to the accessibility of psychological treatment, as more patients can be treated with the same human resources, which is crucial given the low proportion of patients who currently receive CBTp (Burgess-Barr et al., 2023). Cost-effectiveness studies still need to be conducted, but preliminary findings suggest that VR-CBTp is cost-effective in the short term from a societal perspective (Pot-Kolder et al., 2020). If developments of (partly) automated VR therapies continue, treatment efficiency and cost-effectiveness are likely to increase (Freeman et al., 2022).

Our study had several limitations. First, the number of participants was lower than planned (103 instead of 122). Thus, the study was somewhat underpowered. Second, ESM was missing for 17% of the participants at baseline and almost half at posttreatment and follow-up. The frequency and intensity of assessments seem too burdensome. The same ESM scheme was not problematic in our previous RCT (Pot-Kolder et al., 2018); however, a special device was used, and stronger emphasis on completion was put (e.g. completion was needed for randomization). We do not know the impact of the missing data on our findings and the potential biases it could cause; missing ESM data could be related to both better and worse outcomes. In the case of better outcomes, assessments may have been missed because individuals were engaged in other activities, such as work, leading them to ignore the prompts. On the other hand, missing ESMs related to worse outcomes may be due to being preoccupied with symptoms, lack of motivation, or paranoia toward technology or smartphones. The significant finding on our primary outcome was consistent with most secondary outcomes. For secondary outcomes, the completion rate was 83% for posttreatment and 81% for follow-up. Future trials should carefully consider using ESM as a primary outcome; on the one hand, this method enables ecologically valid data; but on the other hand, using daily diary questionnaires has a high risk of missing data. Finally, our findings may have limited generalizability due to the predominantly Dutch participant pool, which may not reflect populations in non-European or non-Western countries. Strengths of the study included the comparison of VR-CBTp with the gold standard CBTp treatment, a rigorous pragmatic multicenter RCT design providing information on feasibility in ‘real-world’ clinical practice, and high-quality treatment in both groups, evidenced by experienced therapists, adequate training and supervision, and high treatment fidelity.

Over the past decade, several promising VR treatments for patients with psychotic disorders have been developed and tested in pilot studies, qualitative studies, and RCTs (Wiebe et al., 2022). VR-CBTp is currently being investigated in another RCT, also with CBTp as a control treatment condition (Jeppesen et al., 2022). If the results of our RCT are replicated and extended, VR-CBTp may be implemented in routine mental healthcare. To achieve this, actions are needed in all parts of the implementation process (ranging from technical support and training to integration in daily workflow and costs), using theoretical implementation frameworks and integrated implementation strategies at multiple levels.

Future research should investigate whether integrating VR-CBTp with in vivo behavior interventions, such as exposure, might give even better results than the current interventions. Moreover, there is a need to investigate which patients benefit most from VR-CBTp, such as those who are unable or unwilling to engage in in vivo exposures. Until present, only two studies have performed such research for VR-CBTp, showing larger benefits for those with a high level of safety behavior and agoraphobia (Berkhof et al., 2024; Freeman et al., 2022), but no other moderators are yet known.

## Supporting information

van der Stouwe et al. supplementary material van der Stouwe et al. supplementary material

## Supplementary material

The supplementary material for this article can be found at http://doi.org/10.1017/S0033291725100949.
