# Supplementary material for: Virtual-reality cognitive behavior therapy versus cognitive behavior therapy for paranoid ideation: A pragmatic, single-blind, multicenter randomized clinical superiority trial
Source: Psychol Med. 2025 Jul 4;55:e188. doi: 10.1017/S0033291725100949 (PMC12270272; doi:10.1017/S0033291725100949)
Supplement: van der Stouwe et al. supplementary material [file S0033291725100949sup001.docx]

**Supplement 1**

Sensitivity analyses excluding the 10 unblinded cases, revealed similar results for all interview (PSYRATS, SBQ-PD and PSP), except for the safety behaviour avoidance scale, where the interaction at follow-up was now significant with p=0·023 in contrast to a p-value of 0·054 for the full sample.
